# The impact of multisensory integration and perceptual load in virtual reality settings on performance, workload and presence

**DOI:** 10.1038/s41598-021-84196-8

**Published:** 2021-03-01

**Authors:** Matteo Marucci, Gianluca Di Flumeri, Gianluca Borghini, Nicolina Sciaraffa, Michele Scandola, Enea Francesco Pavone, Fabio Babiloni, Viviana Betti, Pietro Aricò

**Affiliations:** 1 Department of Psychology, Sapienza University of Rome, Via dei Marsi 78, 00185 Rome, Italy; 2 Braintrends Ltd, Rome, Italy; 3 IRCCS Fondazione Santa Lucia. Rome, Rome, Italy; 4 Department of Molecular Medicine, Sapienza University of Rome, Rome, Italy; 5 BrainSigns Srl, Via Sesto Celere 7/C, 00152 Rome, Italy; 6 College Computer Science and Technology, Hangzhou Dianzi University, Hangzhou, China; 7 Npsy-Lab.VR, Human Sciences Department, University of Verona, Verona, Italy

**Keywords:** Neuroscience, Human behaviour

## Abstract

Real-world experience is typically multimodal. Evidence indicates that the facilitation in the detection of multisensory stimuli is modulated by the perceptual load, the amount of information involved in the processing of the stimuli. Here, we used a realistic virtual reality environment while concomitantly acquiring Electroencephalography (EEG) and Galvanic Skin Response (GSR) to investigate how multisensory signals impact target detection in two conditions, high and low perceptual load. Different multimodal stimuli (auditory and vibrotactile) were presented, alone or in combination with the visual target. Results showed that only in the high load condition, multisensory stimuli significantly improve performance, compared to visual stimulation alone. Multisensory stimulation also decreases the EEG-based workload. Instead, the perceived workload, according to the “NASA Task Load Index” questionnaire, was reduced only by the trimodal condition (i.e., visual, auditory, tactile). This trimodal stimulation was more effective in enhancing the sense of presence, that is the feeling of being in the virtual environment, compared to the bimodal or unimodal stimulation. Also, we show that in the high load task, the GSR components are higher compared to the low load condition. Finally, the multimodal stimulation (Visual-Audio-Tactile—VAT and Visual-Audio—VA) induced a significant decrease in latency, and a significant increase in the amplitude of the P300 potentials with respect to the unimodal (visual) and visual and tactile bimodal stimulation, suggesting a faster and more effective processing and detection of stimuli if auditory stimulation is included. Overall, these findings provide insights into the relationship between multisensory integration and human behavior and cognition.

## Introduction

Although the visual channel is the major source of information which we rely on to navigate in the external environment, natural stimuli are typically multimodal. Multisensory integration is the process through which the brain combines information from independent, but temporally aligned signals that derive from multiple sensory sources (e.g., vision, auditory) into a coherent representation ^1,2^. The integration of visual and auditory signals that emerges because of this spatiotemporal concurrence enhances neuronal responses^3–5^, the Blood Oxygenation Level Dependent (BOLD signal)^6^ and Event-Related Potentials (ERPs)^7^. This enhanced activation is also reflected at the behavioural and perceptual level. Multimodal stimuli in fact, induce faster and more accurate responses than the summed probability of two unisensory stimuli^8,9^ and a broad-band auditory stimulus significantly enhances the perceived intensity of a visual stimulus ^10^ while task-irrelevant tactile stimulations are found to increase auditory intensity ratings^11^. Also, multisensory signals reduce visual search latencies, not only when spatially informative and colocalized with visual targets^12,13^ but also when task-irrelevant or uninformative^14,15^*.* This means that multisensory integration can enhance the ability to detect a target stimulus.

However, this beneficial effect of the multisensory integration is not always evident. Previous work showed that the facilitation effects of the multisensory integration on attentional capture, that is the facilitation in the detection of target stimuli, seem to partially rely on the stimuli to be attended^7^. In this relationship, an important role is played by the perceptual load^16,17^, defined as the amount of information involved in the processing of the task stimuli ^18^. In fact, even if using a peripheral spatial cueing task, previous findings showed that subjective performance is not particularly benefitting from multisensory integration, compared to the unisensory stimulation. By contrast, under a condition of concurrent visual perceptual load (e.g., Rapid Sequential Visual Presentation task—RSVP) the effectiveness of multimodal stimuli improves in enhancing subjective performance, compared to unisensory signals^17^.

To systematically test whether multimodal stimuli are more effective in capturing attention when the perceptual load is higher, we devised a target detection task in which multisensory stimuli were presented under different conditions of perceptual load. To do so, we used Virtual Reality (VR), a powerful tool to develop realistic scenarios that mimics the multisensory stimulation characterizing real-life conditions. In fact, real-life sensory processing and behaviour are complex and dynamic, involving interactions of many context-dependent stimuli through different sensory modalities.

A growing number of studies highlights that complex and multisensory stimuli under naturalistic stimulus conditions produce highly reliable, selective and time-locked activity in many brain regions compared to artificial and highly controlled stimuli, typically employed in conventional experimental protocols (see^19^). However, naturalistic stimulation is often questioned because of its uncontrolled nature^20^. VR experiments can bridge the gap between the control granted by laboratory experiments and the realism needed for a real-world neuroscientific approach ^21,22^. Indeed, VR allows researchers to maintain a high degree of control on the experiment, while at the same time immersing participants in highly realistic multisensory environments^23^. This approach can also help to overcome the limitations of previous studies that failed to shed light on the modulatory effects of load on multisensory stimuli using a silent and dark laboratory environment or loading only the visual channel, as previously remarked by other authors^24^^.^

Despite participants knowing in advance that a virtual scenario is a computer-generated world and is not real, they think, feel and behave as if the events were happening. This phenomenon is at the root of the concept of “*presence*”^25^. The strong physiological and emotional responses can be quantified by means of heart and respiration rate, skin sweating, skin temperature and EEG activity. On the other hand, presence is highly dependent on degree of realism and *immersiveness*^26–28^ i.e., the system’s capability to generate sensory inputs that closely mimic those picked by our senses in a real world environment. In this study, different multimodal signals (e.g., auditory and vibro-tactile) were presented, alone or in combination, concurrently with the appearance of the visual target (i.e., spheres) to obtain different multimodal sensory stimulations. Furthermore, the introduction of perceptually corresponding environmental noise, together with frequent targets, induced a condition of enhanced perceptual load with respect to the low load condition. Also, during the experiment, we recorded Electroencephalography (EEG) signal and Galvanic Skin Response (GSR) along with subjective measures to assess the perceived workload and the sense of presence. The electrodermal response permits to assess the level of arousal: previous studies demonstrated its utility as an objective measure of anxiety under threatening situations or of body illusions^29–31^. Through EEG measures, we extracted features (i) in the time domain (i.e., ERPs analysis, in particular P300 ERPs) and (ii) in the frequency domain (i.e., EEG frequency bands), both modulated the difficulty of the task variations^32–34^.

For the first time, here we investigated the facilitation effect of multisensory integration using a naturalistic target detection task in VR by exploring whether and how auditory and vibro-tactile stimuli (alone or combined) presented concurrently with the visual targets in two different conditions of perceptual load (e.g., low and high) could (i) improve the participants’ detection performance, (ii) enhance their sense of presence in the virtual environment, (iii) modulate the mental workload and iv) enhance the processing and detection of environmental stimuli.

## Materials and methods

### Participants

Eighteen healthy male volunteers (mean ± SD, 26.5 ± 3.2 years old) without significant psychiatric or neurologic diseases and with normal or corrected-to-normal visual acuity participated in the study. We chose to involve only male subjects to reduce the inter-subjects’ variability due to putative gender effects. The experimental protocol was approved by the ethics committee of Sapienza University of Rome and was carried out under the ethical standards of the Declaration of Helsinki of 1964. All participants gave their written and signed informed consent before the experiment.

### Experimental setup

**Figure 1:**
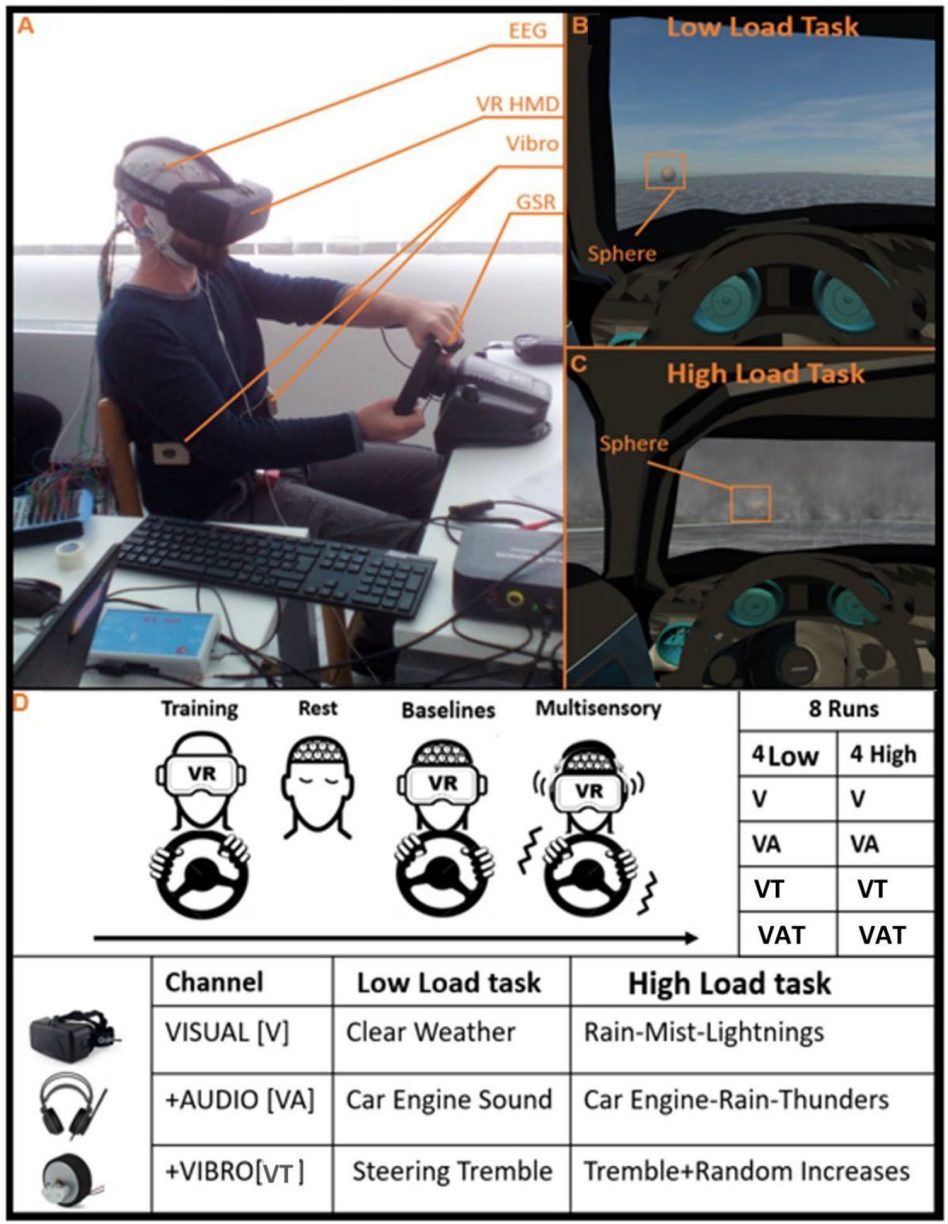
(**A**) Experimental setup; (**B**) the first-person perspective of the low and (**C**) the high load conditions. The target object (the sphere) is shown in the insert; (**D**) summary of the experimental procedure with the details for each task. Informed consent was obtained for this image.

Figure 1A depicts the experimental setup. A virtual scenario consisting of a car on a racetrack was designed in 3ds Max 2015 (Autodesk Inc.) and implemented in Unity3d 5.3.1 (https://unity3d.com). Subjects explored the Virtual Reality (VR) environment from the driver’s first-person perspective employing an Oculus Rift Dk2 head-mounted display (HMD) (www.oculus.com). The HMD has a 100° field of view, a resolution of 960 × 1080 per eye and internal sensors to track head movements. To control the car, a Logitech g27 racing wheel with no shifter was used. Participants were instructed to use only the throttle pedal and to maintain it pressed despite the speed of the virtual car being fixed to reduce the performance variability across subjects. The vibrotactile feedback was delivered using two DC vibrating motors applied to a wearable belt, developed by the Braintrends company (www.braintrends.it) for the experiment purposes. The audio feedback was delivered using headphones. To record skin sweating activity, GSR was monitored utilizing the NeXus-10 MKII system (MindMedia BV, Netherlands, www.mindmedia.com) and its dedicated software BioTrace + . GSR sensors were applied on the index and middle fingers of the participant's non-dominant hand ^35^. The EEG recordings were carried out using 38 channels. The amplifier adopted in the experiment was the Galileo BEPlus (EBNeuro Spa, Italy, www.EBNeuro.biz)*.*

### Experimental procedure

Before the experiment, participants were asked to get familiar with the steering wheel and the pedals. The familiarization procedure was performed in the VR setting during a driving task similar to that employed in the experimental protocol, without any other task to perform. The familiarization procedure was considered completed when participants completed a lap on the virtual racetrack and felt comfortable with the Head Mounted Display^36^. Subjects began the experiment with two 1 min runs in which they were instructed to rest, respectively with eyes open and closed. The eyes closed condition was useful to estimate the alpha peak, i.e. the Individual Alpha Frequency (IAF)^37^, for the frequency-domain analysis (see methods section for more details). This procedure, well established in scientific literature, allows to overcome inter-individual brain rhythms variability by defining individual spectral ranges as a function of the Individual Alpha Frequency itself^38,39^. Then, two baseline conditions were used to calibrate the EEG-based algorithms (see below for further details), while subjects were instructed to drive the car around a racetrack in the virtual scenario, under conditions of low (baslow) and high (bashigh) perceptual load. In the low load condition, the visibility was high with sunny weather, whereas in the high perceptual load the presence of mist and rainy weather with thunder decreased visibility and increased environmental noise. Actually, in several previous works the load of the experimental task has been modulated also through additional tasks (e.g., N-back memory task, mathematical operations, etc.)^40,41^, however such an experimental design would have been far from realistic. In this work the aim was to induce different perceptual demands by modulating external variables directly related to the driving experience, such as the weather. This strategy has been shown to be effective by previous studies^42,43^.

The same two high and low load conditions were also employed in the multisensory experimental task where, this time, subjects were instructed to drive the car while hitting slightly transparent sphere-like objects, spawned randomly on the left or right side of the track. The goal of the task was to achieve the highest possible score by gathering as many targets possible while completing a lap as fast as possible. Figure 1B,C illustrate examples taken from the multisensory task, under low and high load conditions, respectively. The numerosity and the rate of presentation of the spheres, despite being fixed within the multisensory task blocks, was higher in the high load condition (i.e., n = 40), as compared to the low load one (n = 20), in order to consume more attentional resources and increase the task difficulty, as in previous studies (e.g., ^44^). In both conditions, the collectible items could arise from the ground or fall from the sky; on the horizontal plane (i.e. right or left), the appearance was randomized to avoid expectations or habituation effects.

For each load condition, each subject performed 4 runs, 3 min long each, which differed in terms of the sensory channels enabled: *V* for just the Visual channel, *VA* for Visual and Acoustical channels, *VT* for Visual and Tactile ones, *VAT* with all of them. In the V task, participants experienced just visual stimuli, and they could not feel any sound or vibration from the surrounding environment. In the VA task, the Acoustic channel provided a *“beep”* in a stereo mode concomitantly to the spheres’ appearance (providing also information about the side, right or left). Participants could also hear the engine of the car running and, for the high load condition, the sound of the rain and thunder. In the VAT task, subjects were guided towards the stimuli also by the stereo vibrotactile feedback provided through a vibrating belt, tied to their abdomen. The belt consisted in a pair of DC vibromotors, embedded in two wooden cases (i.e., one for each side of the trunk) and produced a vibration, fixed in power and duration, on the left or on the right side in accordance with the spheres position concurrent with the appearance of the target. In this task, also the steering wheel vibrated following the car engine modulation and, for the high load condition, due to the presence of thunder. Because the multisensory integration mimics naturalistic stimulus conditions, the auditory and vibrotactile feedbacks were used concomitantly with the appearance of the target sphere. Figure 1D provides a summary of the employed multimodal perceptual task, for both low and high load conditions.

At the end of each task, the HMD was removed from subjects’ heads to avoid any possible sense of nausea and to allow them to fill the workload and the 3 items questionnaire on the sense of presence, both described in the next paragraph. Subjects started with the low or high load condition in a counterbalanced order. Within each condition, the 4 runs of multisensory stimulation were presented in a counterbalanced way across participants.

### Subjective evaluation

After each experimental run, subjects gave their answer regarding the sense of presence and workload by filling in specific questionnaires (Table 1). The sense of presence questionnaire was adapted from a previous work^29^ and consisted of three questions scored on a scale from 1 to 7. To achieve a final presence score, we averaged the three answers. The NASA Task Load Index (NASA TLX) questionnaire^45^ assesses workload based on 6 subscales. They are rated for each task within a 100-points range with 5-point steps. These ratings were then combined for each Task/Condition to obtain a workload index.

**Table 1:** Questions assessing the perceived feeling of presence and workload.

**Presence**	
Question 1	How much did you get the feeling of being inside the racetrack?
Question 2	How much did you get the feeling that the virtual experience was reality for you and you almost forgot the real world of the lab?
Question 3	When you think back to this experience, do you think of the virtual track as an image you saw or like a place you visited?
**Workload**	
Mental Demand	How mentally demanding was the task?
Physical Demand	How physically demanding was the task?
Temporal Demand	How hurried or rushed was the pace of the task?
Performance	How successful were you in accomplishing what you were asked to do?
Effort	How hard did you have to work to accomplish your level of performance?
Frustration	How insecure, discouraged, irritated, stressed and annoyed were you?

### Neurophysiological measurements

#### EEG recordings

For each subject, scalp EEG signals have been recorded by the digital monitoring *BEplus* system (EBNeuro system, Italy) with a sampling frequency of 256 Hz by 38 Ag/AgCl passive wet electrodes covering all the scalp sites (Fp1, Fpz, Fp2, F3, F4, F7, Fz, F8, AF3, AF4, AF7, AF8, FC3, FC4, FCz, C3, Cz, C4, T3, T4, CPz, C5, C6, CP3, CP4, P3, Pz, P4, P7, P8, PO7, PO8, PO3, PO4, O1, Oz, O2, POz) referenced to both the earlobes and grounded to the AFz electrode, according to the 10–20 standard^46^. In addition, both vertical and horizontal EOG signals have been recorded concurrently with the EEG, and with the same sampling frequency (256 Hz), by two bipolar channels surrounding the right eye, in order to collect the eye-related activity (i.e. eye blinks and saccades) of the subjects during the execution of the task. Two different analyses have been performed to evaluate the experienced workload of the subject across the different Sensory Tasks and Load Conditions.

#### Time-domain analysis

The first analysis has been performed in the time domain, by using Event-Related Potentials (ERPs) elicited by the hit targets. The synchronization between the EEG recording system and the target appearance has been achieved by using a dedicated Trigger Station (BrainTrends, www.braintrends.it).

ERPs represent the EEG voltage fluctuations that are associated in time with some physical or mental occurrences^47^. Specifically, the *P300* event-related potential is a positive deflection of the EEG signal elicited at around 300 ms after the occurrence of a stimulus that the user is paying attention to^48,49^. This component is normally evaluated in terms of amplitude (in µV) and latency (in ms). Although P300 amplitude and latency could be affected by several factors (e.g., attention, fatigue, age, gender)^50^, it has received much attention as a potential indicator of mental workload^51,52^. It is assumed an endogenous potential, as its occurrence links not to the physical attributes of a stimulus, but to a person's reaction to it. For this reason, we employed this kind of analysis to assess how the multimodal integration of sensory modalities could affect the processing and the detection of environmental stimuli. In particular, P300 latency is thought to reflect stimulus classification speed, such that it serves as a temporal measure of neural activity underlying attention allocation and immediate memory operations^53–55^ and the amplitude of the P300 is proportional to the amount of attentional resources engaged in processing a given stimulus^56^. In this study, for each subject we calculated P300 amplitude and latency values, averaged for all the hit targets, for each condition (2 load conditions and 4 sensory tasks). In order to avoid possible biases between low and high load conditions, we averaged the same number of P300 related targets for both the conditions. In particular, for the high load related data, we randomly selected for each subject and for each sensory modality condition 20 target epochs (the number of targets of low load condition). In the following, the algorithm steps to calculate P300 amplitude and latency starting from recorded EEG signals were reported.

Both the EEG and EOG signals have been firstly band-pass filtered with a fifth-order Butterworth filter (low-pass filter cut-off frequency: 30 Hz, high-pass filter cut-off frequency: 1 Hz). Independent Components Analysis (ICA)^57^ has been performed to remove eye blinks and eye saccades artifact contributions that could affect the morphology of the evoked P300 potentials. After that, the EEG signals have been segmented in epochs of 800 ms starting from the onset of the target appearance. For other sources of artifacts (e.g., bio amplifier saturation, muscular activity) specific procedures of the EEGLAB toolbox have been used, in particular the “Threshold”, “Trend” and “Sample-to-sample difference” criteria^58,59^.

None of the epochs related to the targets used for the ERP analysis have been removed, as they were not contaminated by artifacts, except for one epoch, in one subject. Then, the target related epochs have been filtered by using a method described in Aricò et al.^52^ based on the use of wavelet transformation and of the cumulative distribution function, to increase the signal to noise ratio (SNR) of the P300 potentials recorded during the experimental tasks. This algorithm was necessary to compute as accurately as possible amplitude and latency of P300 potentials, starting from a not too high number of stimuli (i.e. 20 targets).

The P300 amplitude is defined as the difference between the largest positive-going peak of the ERP waveform within a time window (e.g., 250–500 ms) and the average of the time-epoch voltage chosen for the analysis (e.g., 0–800 ms). Latency is defined as the time from stimulus onset to the point of maximum positive amplitude within the same time windows. Amplitude and latency studies on P300 potential are normally performed on the Cz electrode, where such a component assumes the higher amplitude^52^. For this reason, only P300 epochs on this electrode have been considered for further analyses. Both in terms of amplitude and latency, the P300 component has been identified and extracted by using a semi-automatic procedure based on the continuous wavelet transform (CWT)^52^. This procedure allowed us to visually check the presence of the P3 waveform, enhance the single trial Signal to Noise Ratio and, finally, to automatically extract single trial P3 potentials. Filtered epochs have been averaged for each subject, load condition, and sensory modality condition (see Fig. 4A). The average of the time-epoch voltage chosen for the analysis (i.e. 0-800 ms) represented the baseline for the ERP analysis.

#### Frequency-domain analysis

The second kind of analysis has been performed in the EEG frequency domain. Concerning the former analysis (ERPs-based), that was strictly locked to the processing of the appearance of targets, such analysis in frequency domain was employed to highlight the overall workload experienced by the subjects along the whole running lap, for each experimental condition. In this regard, most of the studies showed that the brain electrical activities mainly involved in the mental workload analysis are the theta and alpha brain rhythms typically gathered from the *Pre-Frontal Cortex* (PFC) and the *Posterior Parietal Cortex* (PPC) regions. Previous studies demonstrated that the EEG theta rhythm over the PFC presents a positive correlation with the mental workload^60,61^. Moreover, published literature stressed the inverse correlation between the EEG power in the alpha frequency band over the PPC and the mental workload^62,63^. Only a few studies have reported significant results about the modulation of the EEG power in other frequency bands, i.e. the delta, beta and gamma^64,65^. More specifically, most of the studies are focalized on the EEG power modulation occurring in theta (4–8 Hz) and alpha (8–12 Hz) frequency bands, usually associated with cognitive processes such as working memory and attention, typically involved in mental workload. Mental workload is also known to suppress EEG alpha rhythm and to increase theta rhythm during the information encoding and retrieval^66^. Depending on such evidence, theta EEG rhythms over frontal sites, and alpha EEG rhythms over parietal sites have been used for such kinds of analysis. As for the time domain analysis, all the processing and artifact removing algorithms have been applied to i) avoid eye blinks and saccades, that could create a disturbance within the same frequency bands related to workload and cause a bias, and ii) remove all the other sources of artifacts. Regarding the latter, on average the 10% of the EEG signal has been marked as an artifact, and then removed from the analysis. The EEG signal has been segmented into epochs of 2 s, shifted of 0.125 s^31^. The *Power Spectral Density* (PSD) was calculated for each EEG epoch using a Hanning window of the same length of the considered epoch (2 s length that means 0.5 Hz of frequency resolution). Then, the EEG frequency bands of interest have been defined for each subject by the estimation of the *Individual Alpha Frequency* (IAF) value^37,67^.

To have a precise estimation of the alpha peak and, hence of the IAF, as stated before the subjects have been asked to keep their eyes closed for a minute before starting with the experiments. Finally, a spectral features matrix (EEG channels x Frequency bins) has been obtained in the frequency bands directly correlated to the mental workload. In particular, only the theta rhythm (IAF-6 ÷ IAF-2), over the EEG frontal channels (F3, F4, F7, Fz and F8), and the alpha rhythm (IAF-2 ÷ IAF + 2), over the EEG parietal channels (P3, Pz, P4, P7, P8) have been considered as variables for the mental workload evaluation.

#### Workload assessment: the EEG workload index

To select the subjective discriminant EEG spectral features related to the workload, a linear classification algorithm (*automatic stop StepWise Linear Discriminant Analysis*—asSWLDA^68^) has been used. This algorithm has been already validated and successfully employed for EEG-based workload assessment in operational environments, such as air traffic management^31^ and car driving^32^. Once trained with specific “calibration data”, the algorithm can be used to compute a workload index (i.e., W_EEG_ index) on other data by combining the selected EEG features with specific weights in output from the model itself. In particular, for each load condition, the classifier has been calibrated by using the baseline data (i.e. baslow or bashigh and the respective 1^st^ half of the “V” Task (i.e. V low or V high)). We used just such a task (i.e., V) to train the algorithm because the baseline data (i.e. baslow or bashigh) have also been performed without any sensory input except the visual one, like for the V task. In other words, in this way we were sure to use two conditions which differed just for the workload (in fact in the baseline condition there were no targets to collect, in the respective V task the “environmental conditions” were the same but the driver had to hit as many targets as possible), and not because of the presence of other sensory modalities. At this point, we calculated the W_EEG_ index for the 2^nd^ half of each condition, for each load condition (i.e. V low, VA low, VAT low, VT low or V high, VA high, VAT high, VT high), and vice versa (i.e. calibration on 2^nd^ half and W_EEG_ evaluation on the 1^st^ half). Of course, W_EEG_ index over low and High related Conditions was not comparable among load conditions, because of the different normalization. In conclusion, z-score transformation^69^, has been used to compute a normalization of W_EEG_ index distribution.

### Arousal assessment

The signal related to the Skin Conductance, named hereafter Galvanic Skin Response (GSR), has been recorded with a sampling frequency of 64 Hz through the NeXus-10 MKII device (MindMedia BV, Netherlands), a high-quality device consisting of a wireless amplifier with specific GSR sensors applied to the non-dominant hand^34^: by means of two electrodes on the first phalanx of the index and middle fingers, a constant potential is applied in order to induce a skin electrical current. The variations of such current are functions of the skin conductance variations.

The recorded signal was then entirely processed by using the MATLAB software. First, the signal was downsampled to 32 Hz, in order to reduce the data amount. Secondly, the signal was filtered through a 5^th^ order Butterworth low-pass filter, with the cut-off frequency at 2 Hz, in order to remove all the higher frequency components that are not related to the electrodermal activity, such as artifacts due to movements and fast high pressure on the electrode–skin contact. In particular, the latter causes fast and short signal peaks over the GSR signal: in this case a double-step procedure (automatic detection and expert visual check) has been implemented in order to recognize the artifactual transient and to correct it by interpolating the corresponding signal through a piecewise cubic spline^70^. Then, the signal was processed by using the Ledalab suite, a specific open-source toolbox implemented within MATLAB for the GSR processing ( visit the web site for further information): the Continuous Decomposition Analysis^71^ has been applied in order to separate the Tonic (Skin Conductance Level—SCL) and the Phasic (Skin Conductance Response—SCR) components of the GSR. In this regard, Boucsein^34^ provided the most exhaustive review of GSR physiological interpretation and GSR analysis techniques. Briefly, there are two major components for GSR analysis: the SCL (tonic component) is a slowly changing part of the GSR signal, mostly related with the global arousal of a subject during a situation, whilst the SCR (phasic component) is the fast-changing part of the GSR signal, which occurs in relation to single stimuli reactions.

In the following analysis, the mean value of the SCL and the mean amplitude of the SCR peaks during the experimental conditions have been investigated. With respect to the latter, a stimulus-related peak discrimination has not been performed, but all the detected peaks have been considered, since we did not aim at evaluating the physiological response induced by the target, but the overall physiological activation induced by the whole experience. In this sense, higher input frequency during the high load condition could have acted as a confounding factor, therefore only the amplitude mean of these peaks has been considered, in order to be independent from the frequency. Also, in this case, z-score transformation^69^ has been used to compute a normalization of GSR-related measures distribution for each subject.

### Performance assessment

At the end of each run, our software produced a log-file with information related to the number of hit targets (#HitT) and to the total time duration (TimeD). Such information has been collected for each subject and experimental condition. A task performance index has been calculated by combining #HitT and TimeD, normalized respectively to the total number of targets (#TotT) and the minimum time required to run a complete lap (TimeMin, Eq. 1). The latter has been calculated by dividing the track length by the maximum car speed. Thus, the more that values are smaller than 1 the lower the performance. The lowest performance is 0, the maximum 1.

1 $$\frac{HitT}{{TotT}}* \frac{TimeMin}{{TimeD}}{\text{: Performance}}\; {\text{Index}}$$

### Statistical analysis

Data analysis was performed with R-studio (Version 1.1.463), a free software programming language and software environment for statistical computing (RStudio Team, 2016). Sample size was determined by means of a simulation approach, with an effect size of r = 0.5 on GSR (more conservative than in Tieri et al., 2015a, 2015b). The simulation has been executed with an home-made R script, simulating the fixed effects and the random effects with two conditions and 4 levels of multisensory stimulations. The 1000 simulations started simulating a group of 10 participants, adding 4 participants until 50 participants. Simulated sample size suggested a sample size of 18 participants to achieve Power of 85%. For each fixed factor, we now report the marginal and the conditional R2 (mR2 and cR2, respectively). The former is the R2 effect size concerning the model’s fixed effects only, the latter the effect size concerning the model’s fixed and random effects. These effect sizes were computed by means of “MuMIn” package^72^.

We performed a multilevel mixed linear regression analysis (LMM or “mixed-effects models”^73^) through the package Afex^74^. Unlike traditional statistical methods, LMM can deal with grouped, nested or hierarchical structured data^75^. Furthermore, LMM are statistical models that incorporate both fixed-effects parameters and random effects^76^ and can separately treat the effects caused by the experimental manipulation (fixed effects) and those that were not (random effects)^73^. Importantly for the present experiment, LMM can handle missing data^77^. Here we used mixed-effect models to test how our dependent variables are influenced by the experimental manipulations (i.e., i.e. V for Visual, VA for Visual-Audio, VAT for Visual-Audio-Tactile, VT for visual-tactile) and by the perceptual load condition (i.e., low and high). We adopted a different model for each dependent variable. The variables were: (a) performance; (b) subjective ratings of workload (i.e., Nasa-TLX), (c) sense of presence; (d) EEG-based workload (W_EEG_); (e) skin conductance. As fixed effects, we used the load conditions (high vs. low) and the sensory tasks. The resulting models included the subjects as a random factor (i.e., random intercept) and the random slopes of load condition and sensory tasks over subjects. Model selection was carried out using the “anova” function to perform a Likelihood Ratio Test between models with interaction versus models without interaction, when the difference between these two models was not significant the model without interaction was used. Type III ANOVA function with Wald’s χ^2^ statistics from the “car” package^78^ in R was used to determine the statistical significance of the fixed effects. Post hoc comparisons using the Tukey test were carried out from the “lsmeans” package^79^ in R.

## Results

### Performance

**Figure 2:**
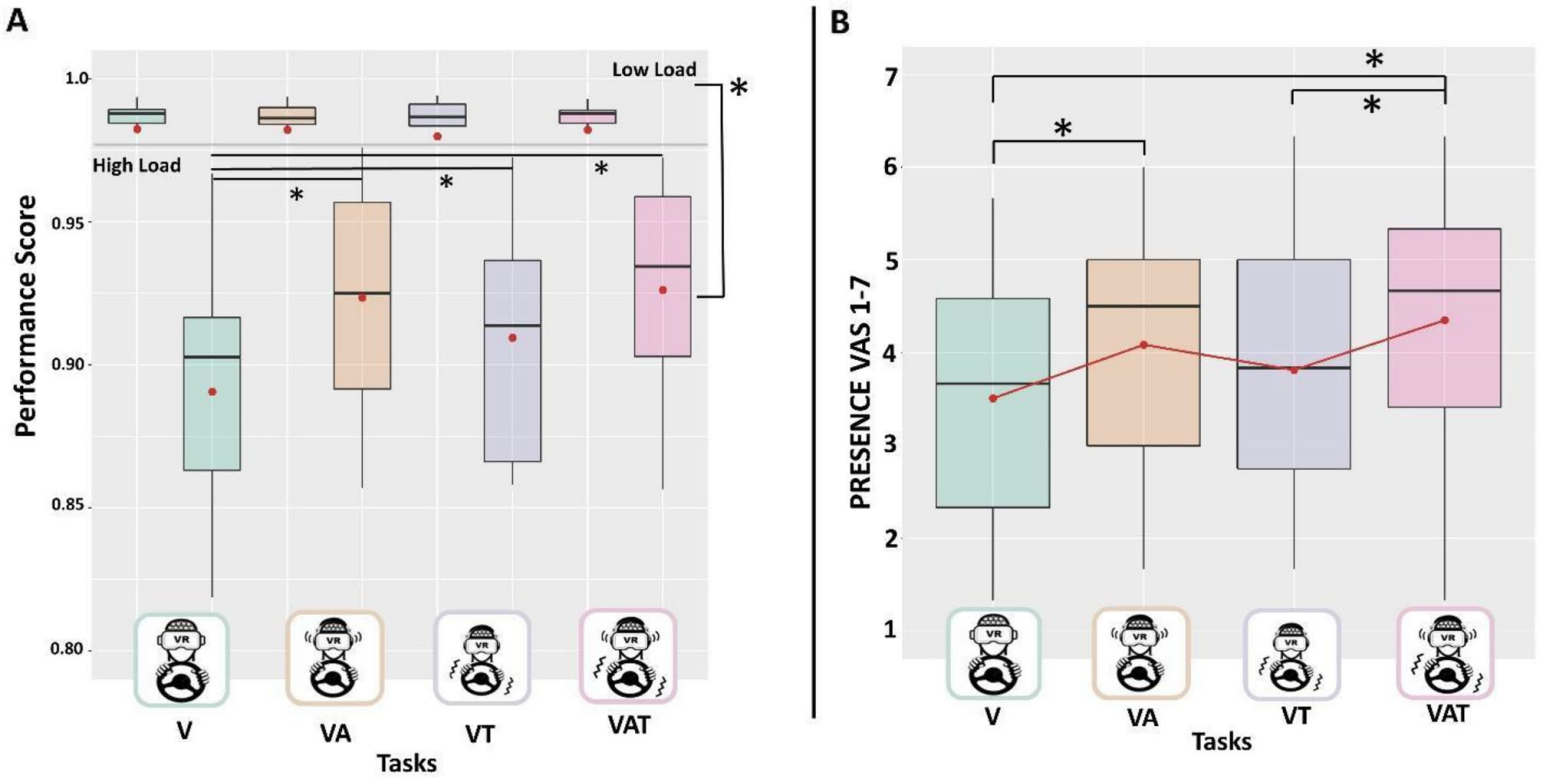
Boxplots representing behavioural correlates of the (**A**) Performance Index for the low load condition (top) and high load condition (bottom) for each sensory task; (**B**) presence questionnaire scores across the sensory tasks. The questionnaire proposed at the end of each run consisted of three items on a scale from 1 to 7. These scores were averaged to obtain a single value for each condition. The black asterisks indicate significant post-hoc tests while red dots and lines refer to the mean. The central line represents the median, the top and the bottom of the box are the first and third quartiles, and the whiskers are the interquartile range of the lower quartile and of the upper quartile multiplied by 1.5.

As a first step, we focused on the effects produced by the multimodal integration and the perceptual load on the subject’s performance. The LMM (marginal R^2^ = 0.53; conditional R^2^ = 0.67) showed a significant main effect of the perceptual load (χ2 (1) < 0.001) explained by reduced performance in the high with respect to the low load condition. Also, we found a significant “ConditionXTask” interaction (χ2 (1) < 0.001) explained by increments of the subject’s performance elicited by both bimodal and trimodal stimulation tasks compared to when only the visual signal is available (VA p = 0.003, VAT p < 0.001, VT p = 0.037), only in the high load condition (Fig. 2A). No significant effect was found for sensory tasks (χ2 (1) > 0.05). No significant effects of sensory tasks and perceptual load nor of their interaction were obtained in the low load condition (all p-values > 0.05).

### Sense of presence

The LMM (marginal R^2^ = 0.10; conditional R^2^ = 0.86) showed the significant main effect of sensory tasks (χ2 (1) = 0.0015) explained by higher sense of presence in the task with higher sensory integration (VAT) with respect to when only the visual modality was available (V) (p = 0.007) or when the auditory feedback was absent (VT) (p = 0.026). The visual and audio integration (i.e., VA) produced a higher sense of presence with respect to the task with the only visual channel (V) available (p = 0.048) (Fig. 2B). Also, we found the significance of the load condition (χ2(1) = 0.0038) explained by a higher sense of presence in the high condition, which increases the sense of presence by β = 0.559 (p = 0.0118) with respect to the low perceptual load condition. The interaction “ConditionXTask” was not significant (χ2 (1) > 0.05).

### P300 analysis

**Figure 3:**
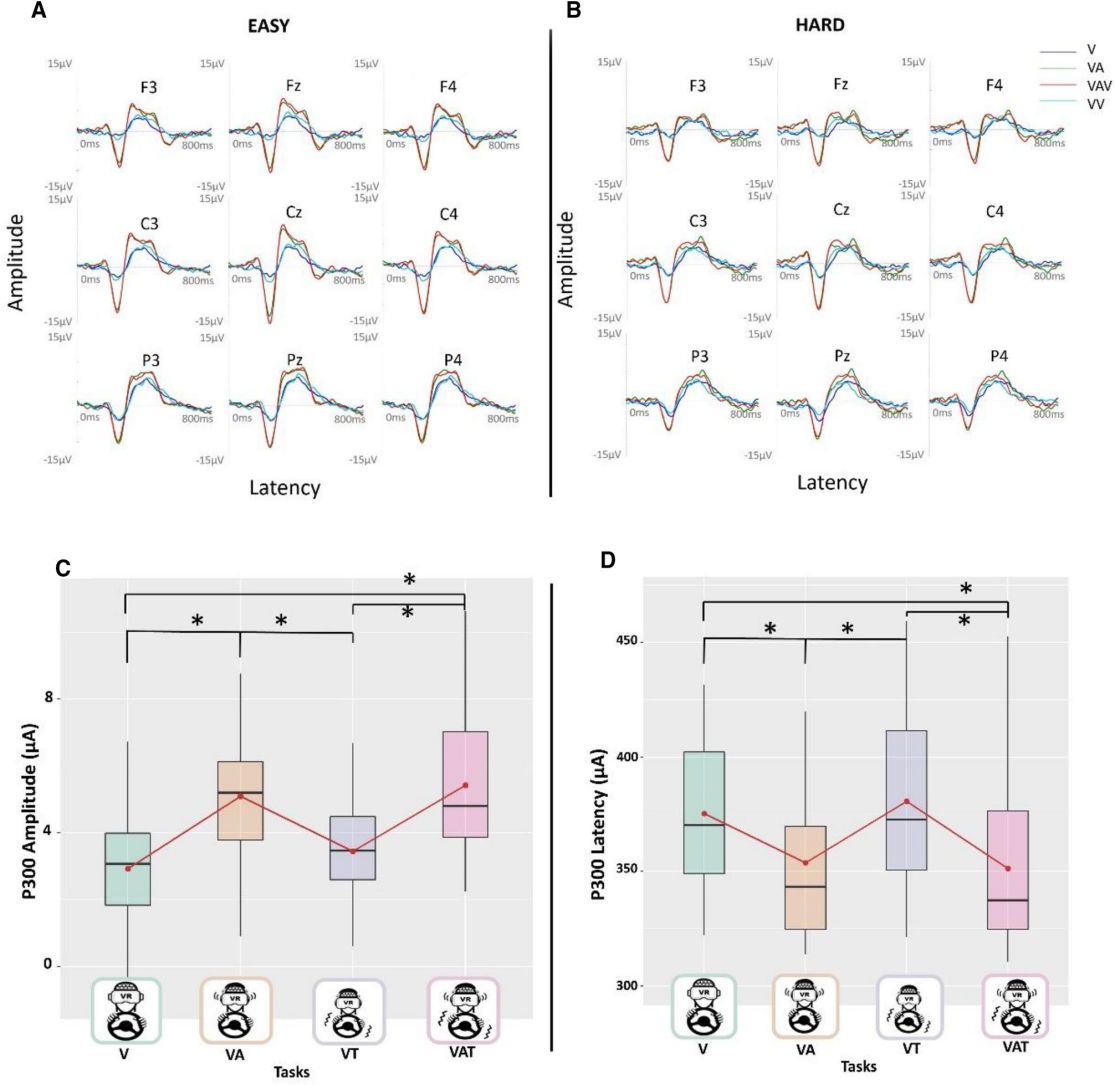
The figures above (**A**, **B**) show respectively the grand average over all the subjects, for each sensory modality and difficulty level, for few representative channels. The figure below shows the significance of the main effect sensory task for the P300 amplitude (**C**) and latency (**D**), low and high conditions have been collapsed over both. The black asterisks indicate significant post-hoc tests while red dots and lines refer to the mean. The central line represents the median, the top and the bottom of the box are the first and third quartiles, and the whiskers are the interquartile range of the lower quartile and of the upper quartile multiplied by 1.5.

Figure 3 shows the P300 amplitude and latency, for the low load (3A) and high perceptual load (3B) conditions. Regarding P300 amplitude, the LMM (marginal R^2^ = 0.31; conditional R^2^ = 0.73) showed the significant main effect of sensory tasks (χ2 (1) < 0.0001). Post-hoc tests revealed an increase in amplitude in both VA and VAT compared to the V task (all p-values < 0.001) and to the VT (all p-values < 0.01) (Fig. 3C). Also, we found a significant “ConditionXTask” interaction (χ2 (1) = 0.01) explained by the VA task being significantly higher in amplitude than the VT task, only in the Low condition (p < 0.001).

For P300 latency, the LMM (marginal R^2^ = 0.26; conditional R^2^ = 0.58) revealed a significant main effect of sensory task and load condition (both p-values < 0.001). The first was explained by an increase in latency in both VA and VAT tasks compared to the V task (respectively p = 0.03 for VA and p = 0.01 for VAT) and the VT (respectively p = 0.02 for VA and p < 0.01 for VAT) (Fig. 3D). The main effect of the Load condition was explained by higher latency in the high load condition (p < 0.001), which increased by β = 0.44 with respect to the low perceptual load. Interaction between load and tasks was not taken into account as the model without interaction didn’t significantly differ from the one with the interaction between the two factors.

### Workload

**Figure 4:**
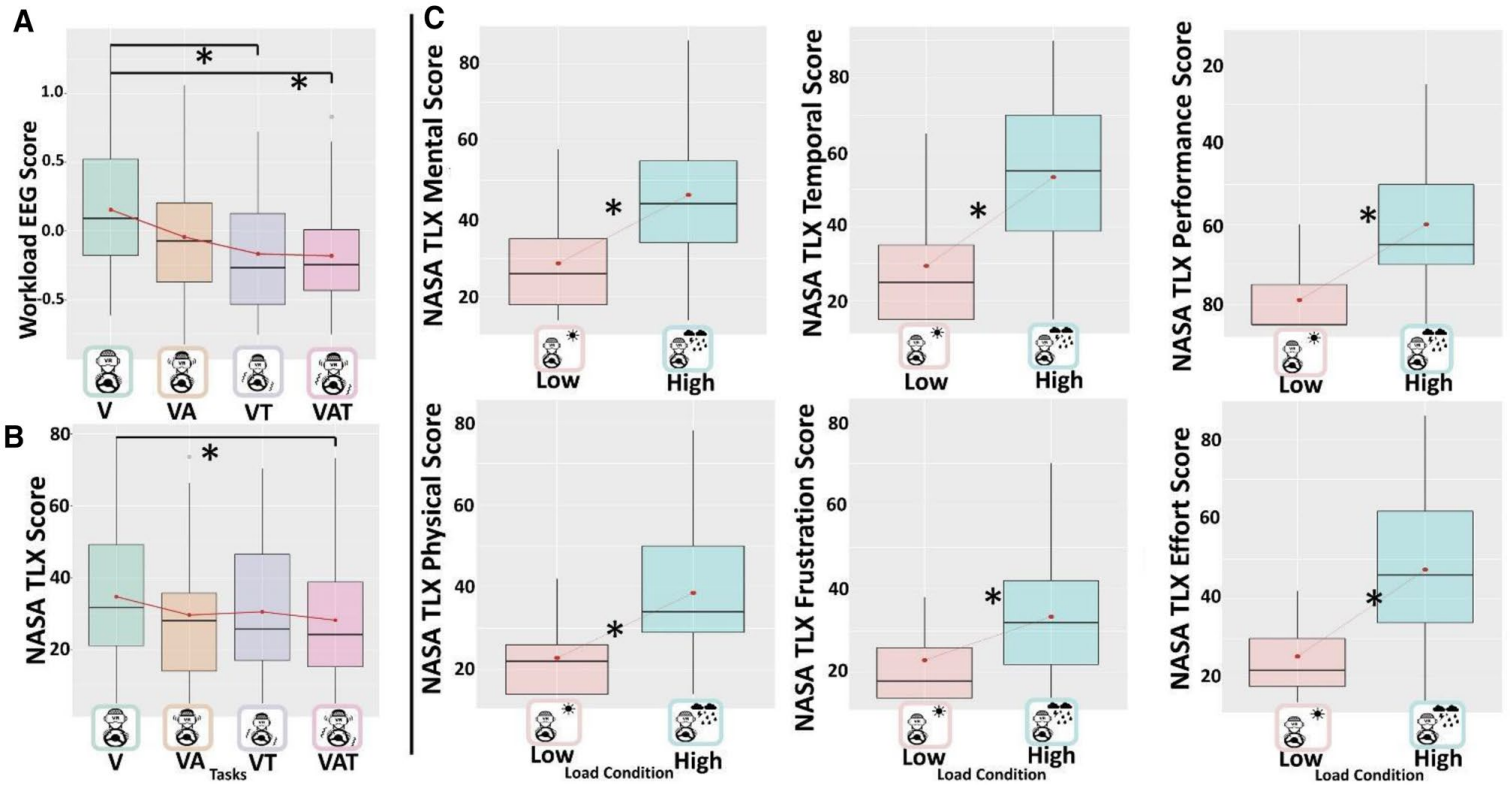
Boxplots representing (**A**) the EEG-based Workload index and (**B**) the workload experienced by the subjects, both across the Sensory Tasks. Panel C represents the different scales of the NASA TLX. The black asterisks indicate significant post-hoc tests while red dots and lines refer to the mean. The central line represents the median, the top and the bottom of the box are the first and third quartiles, and the whiskers are the interquartile range of the lower quartile and of the upper quartile multiplied by 1.5. Performance score scale is inverted (from 100 to 0), coherently with the NASA-TLX questionnaire. In fact, in the workload evaluation, a lower performance means a higher perceived workload.

In terms of EEG-based workload index (W_EEG_), the LMM analysis (marginal R^2^ = 0.08; conditional R^2^ = 0.54) showed the significance of the main effect sensory task (χ2 (1) < 0.001). Post-hoc tests revealed decrement of the workload in the multisensory tasks VAT and VT with respect to tasks in which the only visual channel (V) is available, respectively by β 0.333 (p = 0.002) and by β 0.319 (p = 0.006) (Fig. 4A). No significant effect of the Load Condition or of the interaction with the Sensory Tasks was found (all p-values > 0.05).

Similar effects were found for subjective measures of workload (NASA-TLX) where the LMM (marginal R^2^ = 0.39; conditional R^2^ = 0.79) showed the significant main effects of Sensory Tasks (χ2(1) = 0.015), due again to the reduction of the perceived workload in the task in which the sensory integration is maximal (VAT) with respect to when the visual channel alone is available (V) (p = 0.025) (Fig. 4B). No other comparisons were significant (all p-values > 0.05). Also, we found the significance of the Load Condition (χ2(1) < 0.001), which increases the perceived workload by β = 1.22 to the low Condition. No significant effect of the interaction “ConditionXTask” interaction was found (χ2 (1) > 0.05). Nasa Task Load Index Subscales analyses (Fig. 4C) all showed only the main effect of Load Condition (χ2(1) < 0.001). Across subscales every post hoc test pointed out a significant increase in the High condition compared to the Low one (all ps < 0.001).

### Skin conductance

**Figure 5:**
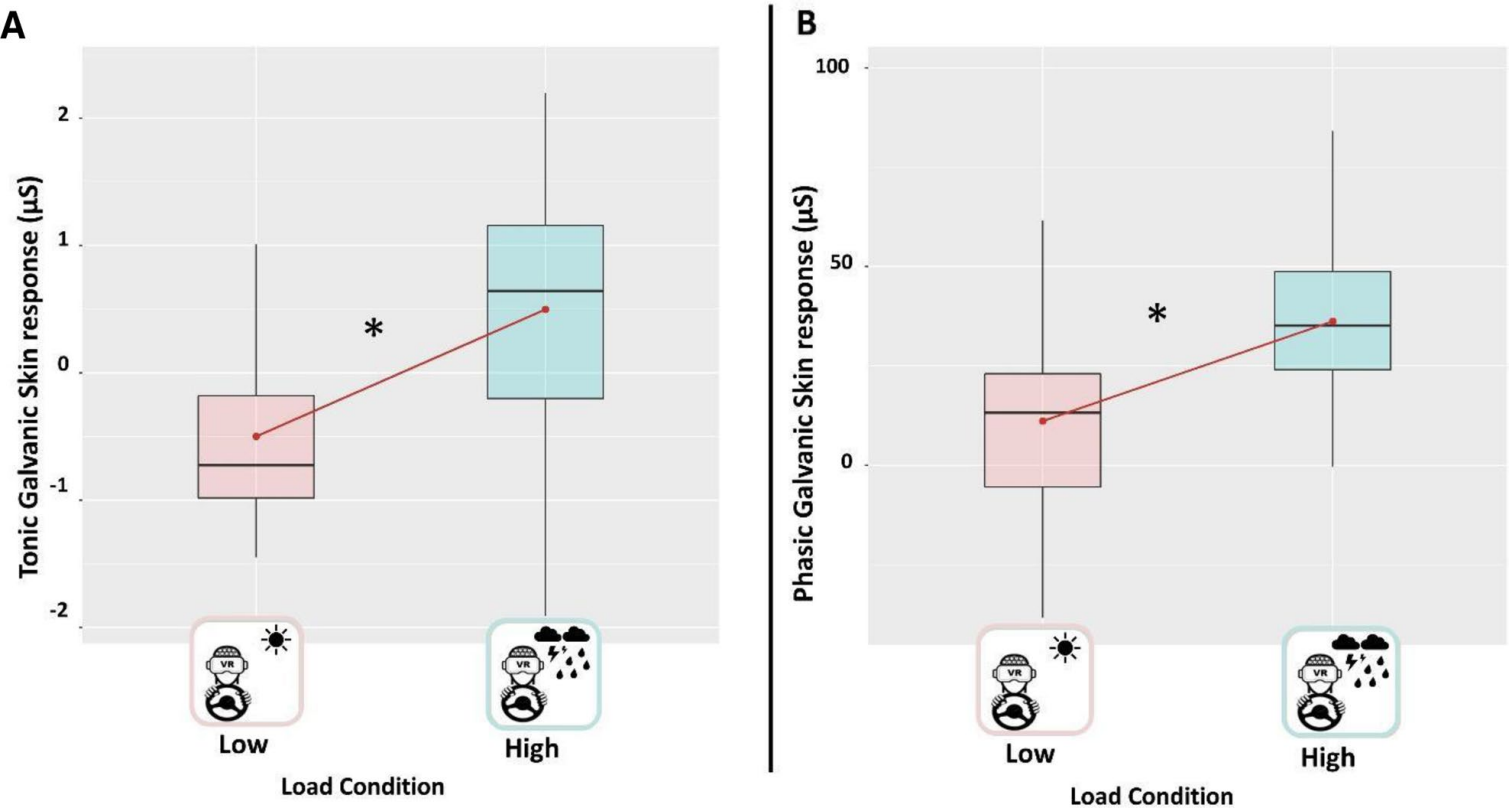
Boxplots representing (**A**) the tonic GSR averaged across sensory tasks for each Load Condition; (**B**) the phasic GSR averaged across sensory tasks for each load condition. The black asterisks indicate significant post-hoc tests while red dots and lines refer to the mean. The central line represents the median, the top and the bottom of the box are the first and third quartiles, and the whiskers are the interquartile range of the lower quartile and of the upper quartile multiplied by 1.5.

Regarding skin conductance, both tonic (marginal R^2^ = 0.26; conditional R^2^ = 0.67) and phasic (marginal R^2^ = 0.25; conditional R^2^ = 0.84) components (i.e., average SCL and SCR peaks amplitude) revealed a significant main effect of the load condition, respectively χ2(1) = 0.001 for the tonic (Fig. 5A) and χ2(1) < 0.001 for the phasic component (Fig. 5B). Post-hoc test revealed higher arousal in the high perceptual load condition with respect to the low load condition (β 0.995, p = 0.004 and β 1.02, p < 0.0001, respectively for tonic and phasic component). A trend toward the significance was found for the main effect Sensory task for the tonic component (p = 0.051). By contrast, this effect was not significant for the phasic component (p = 0.061). Any significant interactions “ConditionXTask” were found, both for tonic and phasic components (all p-values > 0.05).

## Discussion

In this study, we employed a target detection task in immersive virtual reality, neurophysiological signals, and behavioural measures to devise a highly controlled naturalistic paradigm to investigate the facilitation effect of the multisensory signals with respect to the unimodal visual stimulation, under different conditions of perceptual load (high vs low). Our results showed an interaction effect between multisensory integration and perceptual load in enhancing the subjects’ performance, which was higher each time the multisensory integration (bimodal or trimodal) between sensory modalities occurred, but only in the condition of high perceptual load. By contrast, both for the sense of presence and P300 amplitude and latency, the concordance between visual and auditory signals is beneficial, regardless on the delivery of the vibrotactile feedback (VAT and VA, respectively) as compared to the unimodal stimulation. In general, however, the illusion of being present in the virtual environment was higher in the condition of high, not low, perceptual load. By contrast, the haptic feedback was beneficial for the workload, as evident through the EEG-based index and subjective measures (NASA TLX).

### Multisensory integration boosts performance and processing of stimuli

In line with previous reports on the facilitatory orienting effects by multisensory events in different load situations^8,17^, we found that the subjects’ performance in the high perceptual load condition was significantly enhanced by the multimodal stimulation, compared to unisensory stimuli. Differently from classical target detection tasks or spatial orienting tasks in which participants have to press a button or a pedal, here we asked participants to hit the perceived targets by steering a car in a virtual scenario. As can be seen from a reduction in performance, the high perceptual load condition makes the task more difficult compared to the low load condition, causing the subjects to miss more targets. Due to the higher mental load elicited by this condition, both the bimodal (i.e., visual-audio and visual-vibrotactile) and trimodal (VAT) stimulations improve the performance significantly compared to the mere visual condition. This result is confirmed by the P300 analysis, showing an increase in amplitude and a decrease in latency for a multisensory integration (in particular VA and VAT), suggesting a faster and more effective processing and detection of stimuli. These results are in line with the existing literature, showing higher P300 amplitudes and lower latencies of multisensory stimuli (i.e. VA) if compared with unisensory stimulus presentation (i.e. only visual stimuli^80^). In fact, the easier and faster a stimulus is processed, the shorter the P300 latency is expected to be ^53^. Also, the latency jitter of the evoked potentials would be reduced, inducing a larger P300 amplitude^52^. On the contrary, the multimodal integration of vibrotactile modality with the visual one did not play the same “boosting” role of the auditory stimuli. This result is partially in line with the existing literature, showing in particular shorter latencies, but even smaller amplitudes of P300 potentials for auditory relative to vibrotactile stimuli^81,82^. Anyhow, other discrepancies exist in the literature regarding the comparison of P300 effects of auditory and vibrotactile stimuli. For example, Polich and colleagues showed no differences among P300 evoked potentials from vibrotactile and auditory stimuli, by co-varying probability and inter-stimulus interval^83^. In this regard, authors hypothesized the discrepancies in the literature between these findings mainly because of differences in the task settings (e.g., difficulty in the task, concurrence of other tasks). It has to be highlighted also that the perceptual load elicited by the stimuli at the different modalities could be affected by the cognitive load of the main task (i.e., driving task).

Our results converge also with Santangelo and Spence's suggestion on the employment of multisensory stimuli in an applied context^17^. As shown by Ho and colleagues in fact, multisensory stimulation could represent an important feature in the design of driving related signals^84,85^. Additionally, our data extend those results to high load conditions resulting from multisensory environmental noise. However, Lunn and colleagues^8^ demonstrated rather clearly that multisensory stimuli are not ‘immune’ to perceptual load effects as proposed by Santangelo and Spence ^17^ (see also Lavie’s work^86^ on immune stimuli), but they still showed clear evidence of facilitatory attentional capture by multisensory stimuli.

### Workload is decreased by vibrotactile stimuli

To our knowledge, this is the first study exploring the effects of multimodal stimuli on workload through an EEG based index, especially through a virtual reality paradigm. The subjective workload measure assessed by the NASA-TLX^45^ revealed that the trimodal condition was the only one perceived as less demanding compared to the visual one. Conversely, the EEG analysis showed a decrease in workload in both the Visual-Audio-Vibro and the Visual-Vibro condition, regardless of the load (of course, W_EEG_ index over low and high related conditions was not comparable among load levels, because of the different normalization). The reason for this facilitatory effect on the cognitive system could have multiple interpretations. For which concerns the trimodal solution it can be easily conjectured that signals from both audio and haptic feedback do make the task more immersive and easier to perform as one could guess looking at participants' performances (see also^84^), workload and presence questionnaires. Instead, it can be speculated that the absence of environmental sounds, while detrimental for the illusion of presence, for the performance it represents a cognitive relief for those subjects engaged in the task. The mental cost of filtering out the engine sounds and rain noise in the high cognitive load condition, in fact, is probably higher or more stressful^87^ than the one needed to ignore the steering wheel vibrations, and this could explain why, despite the higher presence and performances scorings in the visual-auditory (i.e., VA) condition compared to the visual-vibrotactile one (i.e., VT), the latter happens to be less cognitively exhausting. However, it is worth considering that the different localization between the environmental vibrotactile source (steering wheel) and the cueing source (abdomen) could have contributed to reducing the cognitive effort by facilitating the discrimination of the stimuli from the distracting vibrations in the VT condition. Nevertheless, this finding suggests that in the designing of future workstations, solutions could exploit different sensory combinations to achieve different goals, depending on the task difficulty and duration, and taking into consideration whether to prioritize performances on one side or user stress and mental fatigue on the other.

### Multisensory stimulation and perceptual load modulate the sense of presence

Evidence demonstrates an enhanced sense of presence concurrent with a multisensory exposition in virtual reality^88,89^ A more recent study^90^, carried out with projectors and 3d glasses, describes the relationship between the subjective sense of presence and the combination of sensory modalities (visual-audio-tactile) by reporting an increase of presence in the trimodal condition compared to the bimodal and in the latter compared to the unimodal one.

Our study confirms this claim by using a modern Head Mounted Display. Through a combination of visual, audio and vibrotactile stimulations, accounting for car vibrations, we show that the Visual-Audio (VA) feedback and the Visual-Audio-Vibro one (VAT) are capable of enhancing the sense of presence compared to the visual channel alone (V). In real-life experience, our brain is constantly engaged in the multimodal processing of the surrounding environment. The coordination of two or more sensory modalities facilitates the selection of relevant features from irrelevant stimuli. This explains why the combination of sensorial stimuli elicits a higher sense of presence. However, not all combinations are equally effective. For instance, visual and tactile inputs alone (VT condition) are too far from being a realistic situation to enhance the sense of presence. By contrast, the alignment between the visual and auditory sensory channels more closely mimic real-life conditions. We hypothesize that this effect occurs since the absence of sound represents a highly unconventional situation. Our result is consistent with previous studies reporting that a condition with the absence of sound elicits a lower sense of presence (e.g.,^7,88,89^). Moreover, the lack of acoustical signals could even account for spatial disorientation ^91,92^. Finally, we speculate that the silence to which participants were exposed during the Visual-Vibrotactile task may have contributed to the higher awareness toward the haptic feedback produced by the steering wheel and the belt, which in turn may have introduced an evenly uncommon perceptual situation. Moreover, we found an increase of presence in the high perceptual load condition compared to the low one. In a factor analysis run on the presence questionnaire, Witmer and colleagues^93^ reported that sensory fidelity, immersion, and interface quality accounted for 20.3% of the variance, while involvement alone accounted for 31.9%. From an immersive point of view ^94^, which represents the degree of sensory fidelity achieved by the technological set up^95^ key to evoking a sense of being in the virtual world (see^96^ for review), the two tasks were equivalent. Nevertheless, being in the high load condition more perceptually stimulating and demanding, according to Witmer ^93^ one can speculate that elicited a greater sense of presence because it was more involving.

### Higher perceptual load elicits higher galvanic skin response

The sense of involvement in a given situation is a complex construct to measure since it is a mental state that can involve situational and personal motivation^97^. An increase in skin conductance is considered a reliable bioindicator of human arousal variations^34^ and in virtual reality studies, it is typically used to evaluate the anxiety produced by a situation or the stress related to a threat^26,27,30^. Here we showed that both the phasic and tonic component of the electrodermal response was higher in the high load condition. Peripheral electrodermal responses are found concurrently with the activation of brain regions implicated in emotion, attention, and cognition^98,99^. Despite that we did not directly assess the sense of involvement, we can hypothesize that the high load condition was more arousing and stressful. Higher levels of arousal could have been necessary to the participants to achieve a proper level of performance in a more demanding task and this could have led to an increase in presence.

### Limitations

One possible limitation of the study is that the perceptual load can intrinsically result in enhancing the cognitive load. However, naturalistic stimulation conditions employed in this study did not permit us to disentangle the specific role of the cognitive vs perceptual load and further studies are needed to investigate this issue in a more systematic way.

## Conclusion

Through a virtual reality paradigm and a target detection task, we examined the interplay between the multimodal stimulation and perceptual load on human performance, workload, and sense of presence, by using questionnaires, behavioural measures and neurophysiological measures (i.e. EEG and GSR—based). Five main findings herein are reported.

i. Results highlighted that in virtual reality, in the high perceptual load task only, bimodal and trimodal stimulation were equally effective in significantly improving performance compared to the use of a visual channel alone.
ii. These findings on performances were confirmed by P300 analysis, suggesting a faster and more effective processing and detection of stimuli for a multisensory integration, in particular for VAT and VA, but not for the VT modality.
iii. We showed that the most complete sensory combination (i.e. VAT) and the Visual-Vibro one (i.e., VT) were capable of decreasing the experienced workload according to the EEG-based index. Instead, the perceived workload, according to the NASA-TLX questionnaire, was reduced only by the trimodal condition.
iv. As postulated by Sheridan ^100^ we found that task demand affects the sense of presence, which was higher in the high condition. Moreover, in line with previous studies ^101^, we found that the higher level of multisensory stimulation (e.g., VAT) was more efficient in enhancing the sense of presence compared to the bimodal (VT) or unimodal stimulation (V).
v. We extend previous findings^102^ showing that skin conductance level increases along with the task demand, proving that the same effect occurs in a virtual reality task.

Understanding the most influential factors that moderate the relationship between virtual reality and the human response is crucial for a broad range of studies, ranging from theoretical approaches to perception and cognition to the design of future technological applications and experimental paradigms.

## Data Availability

The datasets generated during and/or analyzed during the current study are available from the corresponding author on reasonable request.
